# Pharmaceutical Strategies for West Nile Virus in Europe, an Underrecognized Cause of Severe Disease and Mortality in Older Adults: From Supportive Care to Antiviral Development

**DOI:** 10.3390/ph19020302

**Published:** 2026-02-11

**Authors:** Luca Soraci, Leonardo Biscetti, Andrea Corsonello, Edlin Villalta Savedra, Guido Gembillo, Filippo Luciani, Alessia Beccacece, Maria Princiotto, Emanuele Nicastri, Laura Ponzetta, Alessandra D’Abramo, Gioberto Filice, Martina Napoli, Maria Elsa Gambuzza

**Affiliations:** 1 Unit of Geriatric Medicine, Italian National Research Center on Aging (IRCCS INRCA), 87100 Cosenza, Italy; a.corsonello@inrca.it; 2 Neurology Unit, Italian National Research Center on Aging (IRCCS INRCA), 60124 Ancona, Italy; l.biscetti@inrca.it; 3 Department of Pharmacy, Health and Nutritional Sciences, University of Calabria, 87036 Rende, Italy; 4 Independent Researcher, 87100 Cosenza, Italy; edlinvillalta@gmail.com; 5 Unit of Nephrology and Dialysis, Department of Clinical and Experimental Medicine, University of Messina, 98122 Messina, Italy; gembillog@unime.it; 6 Infectious Disease Ambulatory, Department of Prevention, Azienda Sanitaria Provinciale, 87100 Cosenza, Italy; filippoluciani@gmail.com; 7 Centre for Biostatistics and Applied Geriatric Clinical Epidemiology, Italian National Research Center on Aging (IRCCS INRCA), 60124 Ancona, Italy; a.beccacece@inrca.it; 8 Centre for Biostatistics and Applied Geriatric Clinical Epidemiology, Italian National Research Center on Aging (IRCCS INRCA), 87100 Cosenza, Italy; m.princiotto@inrca.it; 9 High Isolation Infectious Diseases Unit, National Institute for Infectious Diseases “Lazzaro Spallanzani” IRCCS, 00149 Rome, Italy; emanuele.nicastri@inmi.it (E.N.); laura.ponzetta@inmi.it (L.P.); alessandra.dabramo@inmi.it (A.D.); 10 Independent Researcher, 87100 Cosenza, Italy; giobertofilice@gmail.com; 11 Department of Health Sciences, University Magna Graecia, 88100 Catanzaro, Italy; martina.napoli96@outlook.it; 12 Clinical Pathology Laboratory, Ospedale Ignazio Barone Romeo, 98066 Patti, Italy; gambuzza2002@yahoo.it

**Keywords:** West Nile Virus (WNV), older individuals, Europe, antiviral, vaccine, immunosenescence, interferon

## Abstract

West Nile Virus (WNV) is becoming a significant and enduring public health menace in Europe, propelled by climate changes and accelerated population aging. Most infections are asymptomatic but older adults are more prone to develop neuroinvasive disease, which is characterized by high morbidity and mortality, as well as long-term neurological disturbances and disability. To date, there is still no licensed human vaccine or specific antiviral treatment, and management is mostly supportive. This review brings together the most recent information about WNV epidemiology, pathogenesis, and clinical manifestations, with a special focus on older people in Europe. We critically analyze current and novel pharmaceutical strategies, encompassing drug repurposing, nucleoside analogues, interferon-based therapies, peptides, monoclonal antibodies, and host-directed agents, emphasizing their therapeutic potential alongside the challenges presented by age-related pharmacokinetic and immunological alterations. We also discuss some important gaps in the current evidence base, such as the frequent exclusion of older adults from clinical studies and the lack of a coordinated clinical trial infrastructure that can be quickly activated during seasonal outbreaks. Lastly, we suggest a framework that combines systematic antiviral screening with the creation of a Europe-wide network of clinical trial readiness that is built into current One Health surveillance systems.

## 1. Introduction

West Nile Virus (WNV) is one of the most common neurotropic flaviviruses affecting people and animals worldwide and is spread by Culex mosquitoes [1,2,3,4,5,6]. Originally identified in Uganda, where it was responsible for predominantly sporadic infections in Africa, it has recently transformed into an endemic–epidemic threat worldwide. Climate-driven ecological changes and especially so-called global warming and changes in precipitation patterns have accelerated WNV spread [7]. These phenomena indeed facilitate the proliferation of Culex mosquitoes and reshape avian migratory routes, thereby enhancing viral introduction and persistence in temperate regions [8]. In parallel, population aging is changing the European demographic profile, as individuals aged 65 years and older are more susceptible to WNV infection (WNVI) and are expected to represent more than 30% of Europeans by 2050 [9].

The majority of human infections are asymptomatic; symptomatic cases typically manifest as a self-limiting fever, but in about 1% of cases, infections evolve towards neuroinvasive manifestations such as encephalitis, meningitis, or acute flaccid paralysis, characterized by high morbidity and mortality [10]. Crucially, older and immunocompromised individuals are more commonly affected by neuroinvasive WNV [11,12]; multiple cohort studies have shown that advanced age represents one of the strongest predictors of poor outcomes [11,13,14], including increased hospitalization rates, prolonged length of stay, enhanced disease severity, as well as increased death [1,15,16].

Several age-related physiological changes may increase the vulnerability of older patients to WNVI. On one hand, the age-related immune function decline, known as immunosenescence, basically impairs antiviral responses [17,18,19] through various mechanisms; these mainly include a decrease in interferon (IFN) release, natural killer cell activity, as well as impaired antibody production by lymphocytes or production of antibodies with poor specificity [20,21]. On the other hand, the chronic low-grade inflammatory state known as inflammaging may enhance viral pathogenesis while limiting effective clearance [22,23]. Furthermore, the presence of frailty and multimorbidity may compromise immunological functions in older individuals [16] and increase susceptibility to infections and their complications [24,25,26].

Aging is also associated with incomplete viral clearance and subsequent delayed recovery from WNVI; although chronic sequelae and long-term viral persistence remain incompletely understood, animal models and human case series suggest that delayed neurological recovery and residual deficits are not uncommon [27]. Aging is characterized by disruption of the blood–brain barrier integrity due to a decrease in tight junction expression, with a subsequent increased risk of viral entry into neural tissues [28,29]. Pre-existing age-related neurodegenerative changes may decrease neuronal reserve and limit compensatory mechanisms, thus amplifying the impact of viral encephalitis [30,31]. For similar reasons, once neurological injury occurs, recovery in older adults tends to be slow and often incomplete: many survivors experience persistent weakness, cognitive deficits, movement disorders such as tremors and Parkinsonism, and functional disability [32,33].

In older populations, diagnosis poses additional difficulties. The low sensitivity of early molecular testing in peripheral blood and the high percentage of mild or asymptomatic infections make it difficult to identify WNV cases. While serological testing for WNV-specific IgM and IgG antibodies and the detection of WNV RNA in blood, cerebrospinal fluid (CSF), or urine forms the basis of diagnosis, test performance is highly dependent on the timing of the sampling and the patient’s immune status. Viral RNA can be detected only during early stages of viremia; whole blood offers a longer detection window and superior sensitivity (≈87%) compared to serum or CSF, while RNA may persist longer in urine but with variable sensitivity [34]. Serological testing complements molecular diagnostics: IgM antibodies typically appear between days 3 and 8 after symptom onset and may persist for months or even over a year, while IgG emerges around 2–3 weeks post-infection, with increasing avidity distinguishing recent from past infections [35,36]. However, immunocompromised or older patients may produce delayed or absent IgM responses, reducing serologic sensitivity and necessitating reliance on RNA detection or neutralization testing [37]. Moreover, cross-reactivity among flaviviruses such as Usutu or dengue can lead to false-positive IgG results, further complicating diagnosis [38]. The most accurate serological test for WNV diagnosis is the Plaque Reduction Neutralization Test (PRNT) [39]. It can be carried out using a highly sensitive 50% or less-sensitive 90% endpoint (PRNT_50,_ and PRNT_90_ respectively). The PRNT_50_ is generally used only for diagnosis of subclinical infections and therefore it has a relevant usefulness mainly in the context of epidemiological surveillance programs. The PRNT_90_, instead, is generally used as a confirmatory test for WNVI. However, due to the broad antigenic cross-reactivity between different flaviviruses, both the PRNT_90_ and PRNT_50_ require evaluation of the neutralizing antibodies against a panel of related viruses, like for instance Japanese encephalitis virus, tick-borne encephalitis virus, yellow-fever virus and dengue viruses, based on both clinical and epidemiological considerations. Furthermore, some technical issues limit the generalizability of results obtained by means of the PRNT across different laboratories (e.g., differences in times of incubation), thus rendering the large-scale applicability of the PRNT quite difficult [39]. Therefore, the PRNT for WNVI diagnosis is currently performed only in reference laboratories following rigorous protocols.

Beyond technical difficulties, clinical aspects also represent a relevant challenge in view of WNVI diagnosis, especially when WNV affects older people. In fact, older patients affected by WNV disease often present atypically with delirium, functional decline, or falls rather than classic fever and headache [1,40,41], and therefore are rarely tested for WNVI.

These diagnostic obstacles probably cause a significant underestimation of the true incidence of WNV disease, especially in older populations, and make it more difficult to identify outbreaks in a timely manner.

With nine identified lineages, WNV exhibits significant genetic variation from a virological standpoint, with lineages 1 and 2 being most closely linked to human neuroinvasive illness [42,43]. The fact that both lineages are already established in Europe emphasizes the shift to persistent endemicity on the continent. Unfortunately, no disease modifying therapy or vaccination is currently available for WNV disease [1]. This helps to explain the high fatality rate of neuroinvasive forms of the disease. So far, only supportive care can indeed be used to manage WNV encephalitis.

Scientists are putting more and more effort into finding small-molecule inhibitors, biologics, and host-directed therapies that target important viral structural and nonstructural (NS) proteins. Improvements in medication design and delivery methods, as well as the use of machine learning, artificial intelligence, and multi-omics platforms, have sped up the process of finding possible antiviral candidates and figuring out how viruses and hosts interact [44,45]. There are many problems that need to be solved before translation can be used in the clinic. One of these is the lack of a coordinated trial infrastructure that can be quickly put into place during seasonal outbreaks. A more significant issue is the systematic exclusion or underrepresentation of older people in antiviral clinical studies. Moreover, age-related pharmacokinetic alterations, such as diminished renal function, modified hepatic metabolism, and variations in volume of distribution and adipose tissue, may influence drug clearance; however, these parameters are inadequately defined for the majority of suggested WNV therapies. In this setting, improving pharmacological preparedness for WNV is essential. This review combines what we know about epidemiology, clinical features, and WNV surveillance, with a focus on the European context. This review also talks about current and new treatments for West Nile Virus and lays out a conceptual framework that aims to speed up the development of antiviral drugs. Instead of repeating specific operational proposals, we want to stress how important it is to combine pharmaceutical innovation with public health surveillance in Europe. This kind of integration could help close the gap between discovery and clinical implementation and make us better prepared for WNV outbreaks.

### Literature Search and Review Approach

This review was conducted using a narrative but structured approach, aimed at integrating epidemiological, clinical, and pharmacological evidence on WNVI, with particular focus on older adults and the European context. A comprehensive literature search was performed in PubMed/MEDLINE, Scopus, and Web of Science, covering studies published from January 2000 to December 2024. Additional references were identified through manual screening of reference lists from relevant articles and international reports from public health agencies. Studies were included if they addressed at least one of the following domains: (i) epidemiology of WNV, (ii) clinical manifestations and outcomes, particularly in older adults, (iii) pathogenesis and host immune response, (iv) antiviral or immunomodulatory therapeutic strategies, or (v) vaccine development. Articles not available in English, conference abstracts without full text, and studies not directly relevant to human disease were excluded.

Preclinical evidence (in vitro and animal studies) and clinical evidence (case reports, observational studies, and clinical trials) were evaluated and discussed separately when appropriate to clearly distinguish mechanistic insights from translational or patient-level data. Given the heterogeneity of available evidence and the limited number of randomized clinical trials, a formal systematic review was not considered appropriate; instead, a narrative synthesis was used to contextualize emerging therapeutic strategies within clinical and geriatric medicine perspectives.

## 2. West Nile Virus (WNV): Pathogenesis and Clinical Manifestations

### 2.1. Clinical Manifestations in the General Population and Older Adults

WNV, a member of the Japanese encephalitis antigenic complex within the Flaviviridae family [46], was first isolated in 1937 in the West Nile province of Uganda from a febrile patient [46,47,48]. For many years, WNVI was considered a mild, self-limited disease, with most cases either asymptomatic or presenting with an influenza-like syndrome known as West Nile fever [10]. However, large outbreaks reported over the past two decades in Europe and worldwide have shown a substantial rise in severe neurological infections [49,50].

WNV contains a positive-sense, single-stranded RNA genome enclosed within a spherical envelope and an icosahedral nucleocapsid [6]. The virion polyprotein precursor is hydrolyzed by viral and host proteases, thus generating three structural (S) proteins, namely capsid protein, membrane protein, and envelope protein (Ep), together with seven NS proteins. Both the S and NS viral proteins play a crucial role in the biology and/or the pathogenesis of WNVI [6].

**Figure 1:**
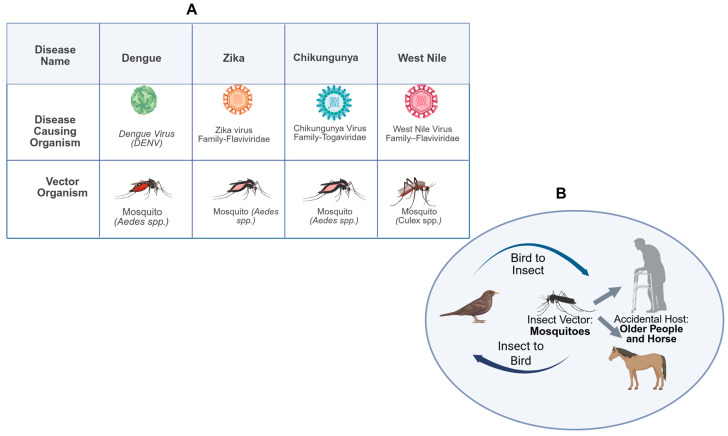
Comparison of major mosquito-borne arboviruses and transmission cycle of WNV. (**A**) Overview of selected mosquito-borne arboviral diseases including dengue, Zika, chikungunya, and West Nile Virus (WNV), highlighting the causative viruses and their principal mosquito vectors. Dengue, Zika, and chikungunya are primarily transmitted by *Aedes* species mosquitoes, whereas West Nile Virus is mainly transmitted by *Culex* species. (**B**) Schematic representation of the enzootic transmission cycle of West Nile Virus, which is maintained between birds and mosquitoes. Humans and horses act as incidental, dead-end hosts and do not contribute to further viral transmission. Older adults are particularly vulnerable to progression from WNV infection (WNVI) to neuroinvasive disease. Created in BioRender. Soraci, L. (2026), https://BioRender.com/st94b8c (accessed on 31 January 2026).

*Culex* mosquitoes and bird reservoirs represent the main drivers of the WNV enzootic transmission cycle, while humans, horses, and other mammals represent incidental “dead-end” hosts [51] (Figure 1).

Once confined to Sub-Saharan Africa, WNV now infects hundreds of millions annually worldwide [52]. Climate-related environmental changes, including milder winters and prolonged warm seasons, have contributed to its expansion; these factors have increased mosquito survival, thus prolonging the period of vital transmission, thereby facilitating the onset of endemic infection in temperate areas [8]. The incubation period in humans following a mosquito bite varies from 2 to 14 days in immunocompetent persons and may extend in older adults or immunocompromised patients. Most infections do not present any symptoms or show up as a short-lived fever [50]; symptomatic infections usually present with fever, malaise, headache, anorexia, myalgia, ocular pain, and gastrointestinal symptoms; a morbilliform rash may be observed in up to 50% of patients during defervescence [53]. About 1% of infections progresses to West Nile neuroinvasive disease (WNND), characterized by high mortality, with a case fatality rate of 10–30% [50]. Older individuals, as well as those with multimorbidity, polypharmacy, and immunosuppressive treatments, are particularly susceptible to WNND [12], which typically manifests with meningitis and encephalitis, characterized by a range of symptoms from mild disorientation to severe encephalopathy, and coma; tremor is frequently reported and accompanied by myoclonus and opsoclonus–myoclonus [50,53]. In older individuals, these movement disorders may be erroneously attributed to pre-existing Parkinson’s disease or other neurodegenerative illnesses, thus postponing appropriate diagnosis [54]. Acute flaccid paralysis, due to invasion of anterior horn cells, is another serious consequence [50,53]. In any case, WNND carries particularly poor prognosis in older adults, as it is characterized by delayed and incomplete recovery and long-term disability [55,56].

Beyond its recognized neuroinvasive manifestations, WNV has also demonstrated significant renal tropism [57], associated with renal focal interstitial lymphocytic infiltration and weakened WNV-specific T lymphocyte response; this compartmental immunity appears to facilitate viral persistence and may induce WNV-related kidney damage. The clinical relevance of these experimental findings became apparent through the Houston cohort. Nolan et al. evaluated 139 participants at 4–9 years post-infection and found that 40% met diagnostic criteria for chronic kidney disease, far exceeding expected prevalence [58]. Notably, neuroinvasive disease history was the sole independent CKD predictor; traditional cardiovascular and metabolic risk factors showed no association, suggesting direct viral pathogenesis. The question of whether this represented true persistent infection rather than residual injury from acute illness was addressed through direct viral detection. Murray’s group found WNV RNA in urine from 20% of patients at 1.6–6.7 years post-infection [27]. Subsequent electron microscopy has revealed that intact 40–60 nm viral particles appeared in sediment from patients up to nine years post-infection [59]. These observations are consistent with broader epidemiological patterns. Patel’s systematic review of 67 studies encompassing >45,000 cases identified chronic kidney disease among the most common long-term sequelae, with risk concentrated in older males with cardiovascular comorbidities [60].

In rare cases, WNVI can be complicated by viral-induced myositis and rhabdomyolysis [12,61], hepatitis and pancreatitis [61]. Very rare but serious complications include myocarditis and multifocal choroiditis, characterized by a negative prognosis [62].

### 2.2. WNV Pathogenesis and Risk Factors for Progression to Neuroinvasive Disease

**Figure 2:**
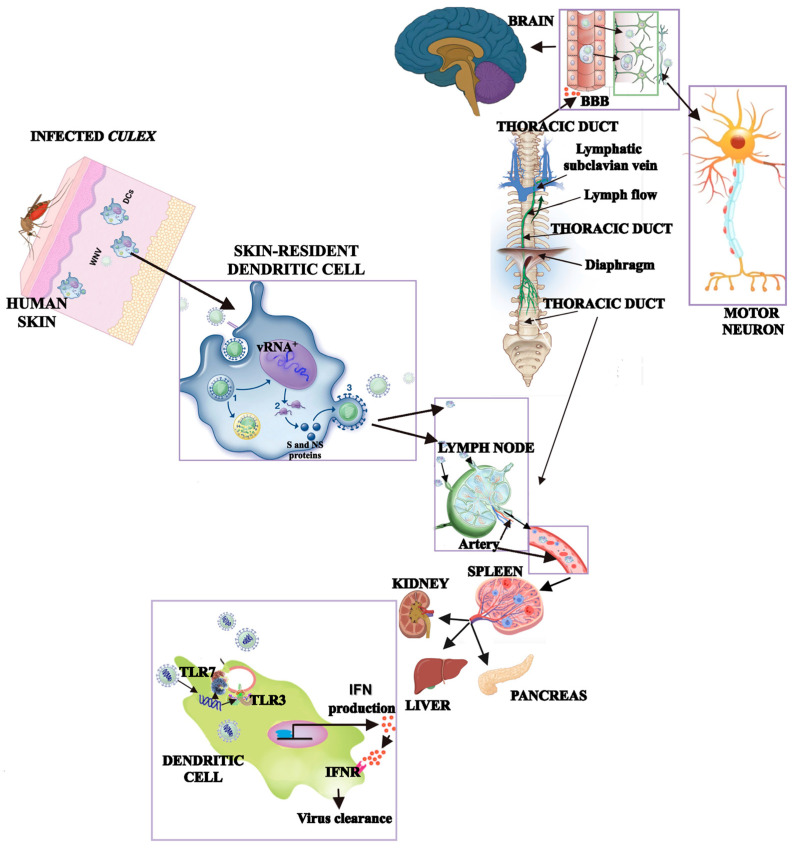
Transmission of WNV. Transmission of West Nile virus (WNV) mainly occurs through the bite of an infected mosquito. Following inoculation into the skin, the virus attaches to keratinocytes and skin-resident dendritic cells through ligand–receptor–mediated endocytosis (step 1), involving dendritic cell–specific C-type lectin receptors, the mannose receptor, and several glycosaminoglycans. Viral particles are subsequently internalized into endosomes, where capsid disassembly allows release of the viral RNA genome into the cytoplasm (step 2). Viral replication and assembly lead to the release of newly formed virions through the secretory pathway as mature infectious particles (step 3). Infected dendritic cells then migrate to regional lymph nodes, where viral replication is amplified and innate immune responses are initiated. Within lymphoid tissues, viral RNA (vRNA) is sensed by endosomal pattern-recognition receptors, particularly Toll-like receptor 7 (TLR 7), triggering type I interferon (IFN) production and inflammatory signaling. Incomplete viral clearance permits dissemination via the lymphatic system and thoracic duct into the bloodstream, resulting in viremia and infection of peripheral organs such as the spleen, liver, kidney, and pancreas. From this systemic phase, WNV may gain access to the central nervous system through multiple, non-mutually exclusive mechanisms, including hematogenous spread with blood–brain barrier (BBB) disruption, transendothelial passage, peripheral nerve invasion, or trafficking of infected immune cells. Once within neural tissues, local innate immune responses critically regulate viral replication and contribute to the heterogeneity of neurological disease severity.

The most common route of human infection by blood-borne WNV is the bite of an infected mosquito (Figure 2). After inoculation into the skin, WNV attaches to the surface of keratinocytes [63] and skin-resident dendritic cells [64] through receptor-mediated endocytosis involving several cellular receptors, including the C-type lectin DC-SIGN, the mannose receptor, and various glycosaminoglycans. These interactions facilitate viral entry and the initiation of replication in epithelial and dendritic cells, which in turn support the internalization of viral particles into endosomes. During endosomal maturation, progressive acidic conformational changes promote capsid dissociation and release of the viral RNA genome into the cytoplasm.

The positive-sense RNA genome is subsequently translated into a single polyprotein that is cleaved by host and viral proteases into three structural proteins (SC, SPrM/M, SE) and seven NS proteins (NS1, NS2A, NS2B, NS3, NS4A, NS4B, NS5), resulting in the expression of the ten viral proteins required for viral replication and assembly (Figure 2). Newly formed virions are transported to the cell surface in exocytic vesicles and released as mature infectious particles [65].

After infection, dendritic cells travel to regional lymph nodes and help the WNV spread throughout the body by getting into the bloodstream and spreading to visceral organs. A few days after infection, WNV can reach the nervous system through many non-mutually exclusive routes, such as peripheral motor neurons, disruption of the blood–brain barrier integrity, the transendothelial passage, and trafficking of infected immune cells into the central nervous system (CNS). Once the virus infects neural tissues, local innate immune responses become crucial, playing a key role in modulating viral replication and influencing disease severity, thus explaining the heterogeneity of neurological outcomes [66].

The RNA genome is replicated and transcribed as a single polyprotein that is cleaved by host and viral proteases into structural and NS proteins. The new virions are released as mature viruses from the host cell surface. Host infected immune cells then migrate to the lymph nodes and, from there, the viral progeny enters the bloodstream via the thoracic duct, from which it is disseminated to visceral organs. The innate immune response, through Toll-like receptors (TLR) 7 and 3 and associated with interferon (IFN) 1 production, eliminates WNV from the peripheral infected organs. WNV can reach the CNS, through multiple pathways, including transsynaptic spread, breakdown of the blood–brain barrier (BBB), transendothelial spread, and infection of innate immune cells that cross into the brain parenchyma. From the CNS, WNV propagates trans-synaptically by axonal transport to motor neurons.

The first host defense mechanism against WNV is represented by activation of innate immune responses: the recognition of viral RNA by Toll-like receptors 7 and 3 triggers the production of type I interferon (IFN-I), which promotes viral clearance from peripheral organs, generally between six and eight days after infection [67]. However, even when peripheral viral loads are low, WNV may continue replicating within the CNS [68,69], where it shows preferential tropism for motor control structures and propagates trans-synaptically through both anterograde and retrograde axonal transport [70].

**Figure 3:**
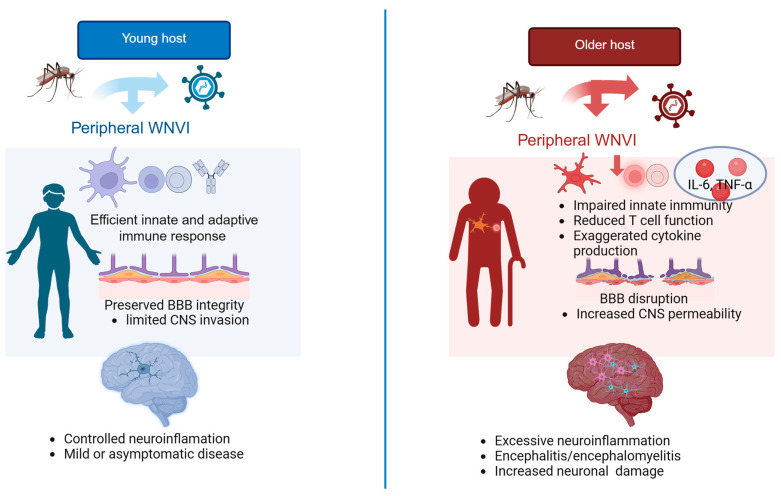
Age-dependent immune responses and neuroinflammatory outcomes following West Nile Virus infection. Left panel: in young hosts, WNV infection (WNVI) triggers an effective innate and adaptive immune response, leading to optimal peripheral viral control and preservation of blood–brain barrier (BBB) integrity. As a consequence, viral entry into the central nervous system (CNS) is limited, neuroinflammation remains controlled, and infection is typically asymptomatic or mild. Right panel: in older hosts, age-related immune dysfunction is characterized by impaired innate immunity, reduced T-cell function, and exaggerated pro-inflammatory cytokine production (including IL-6 and TNF-α). These alterations contribute to BBB disruption and increased CNS permeability, facilitating viral neuroinvasion and progression from WNVI to neuroinvasive disease, which is associated with severe neurological manifestations, such as encephalitis or encephalomyelitis, and increased risk of long-term neuronal damage, disability and mortality. Created in BioRender. Soraci, L. (2026), https://BioRender.com/sdnnz85 (accessed on 31 January 2026).

The onset and severity of neurological symptoms depend on several viral and host-related factors. The specific genetic lineage of WNV strongly influences the progression of WNVI to WNDD, as lineages 1 and 2 are most associated with severe neurological disease in humans among the nine WNV genetic lineages [42,43]. Lineage 7, also known as Koutango virus [71], which is exclusively present in Africa (even if preliminary reports suggests its sporadic presence also in Northern Italy) [72], is currently under intensive investigation, since some studies support its higher virulence compared to other lineages [73] and an increased resistance to interferon due to the presence of a mutation in the NS5 protein of the virus (serine (S) to phenylalanine (F) at the 653rd position in the NS5 protein) [74]. Among the host-related factors, advanced age with its associated immune dysregulation, known as immunosenescence, and chronic comorbidities such as diabetes mellitus and hypertension substantially increase susceptibility to neuroinvasion and worsen clinical outcomes [16,75]. The main differences between young and old individuals in immune responses and neuroinflammatory outcomes are depicted in Figure 3.

### 2.3. WNV Surveillance Mechanisms in Europe

The accurate evaluation of worldwide WNV prevalence is hindered by gaps in epidemiological data, especially from African countries. In Europe, on the other hand, as notification of human and equine WNV infections is mandatory [76], numerous initiatives have been undertaken in recent years to build an accurate surveillance system for this disease. The European Centre for Disease Prevention and Control (ECDC) has put in place a coordinated One Health framework, including the European Commission, Member States, and agencies such as the European Food Safety Authority (EFSA); this framework aims to identify, prevent, and control emerging infectious hazards by considering humans, animals, and the environment as a whole [77]. One of the main goals of human WNV surveillance is the prevention of secondary transmission transfusion-derived blood products [78]. This may lower the number of outbreaks and the costs to society and helps support health security [79,80].

Recent European Union disease reports have underlined the increasing incidence of WNV in Europe in the last years, associated with a marked age-related disease gradient [77,81,82]. As of 3 December 2025, 14 countries in Europe reported 1112 locally acquired human WNV infections, representing a 47% increase compared with the 10-year average of 758 cases [77,81,82]. Ninety-seven patients died. Most infections occurred in men and older individuals [77]; also, fatality rates were significantly higher in older individuals (around 15%) compared to those in younger patients (10%) [2,77,82].

The geographic distribution of WNVI cases showed a particular density in the Mediterranean area; Italy experienced its largest outbreak ever reported since surveillance began in 2008, with 779 cases recorded in 2025, and 72 deaths, accounting for over 70% of human cases in Europe in 2025 [77,82]. Lazio and Campania were the two most affected Italian regions, with 267 and 133 cases respectively. Other countries were Greece (96 cases), France (62 cases), Serbia (62 cases), Romania (49 cases), and Spain (36 cases) [77,81,82].

WNVI-related hospitalization rates were particularly high, with 84% of diagnosed infections requiring hospitalization in 2025 compared to 89% in 2024; this likely reflects the tendency to diagnose severe cases, particularly among older individuals who are more likely to develop symptoms requiring medical attention [2,77,82]. However, it is important to underline that also in Europe, as in the rest of the world, the epidemiological assessment of WNV prevalence and incidence is probably biased by relevant underreporting due to the high number of asymptomatic or paucisymptomatic cases. With respect to the frequency of the neuroinvasive forms of WNV on the total of reported cases in Europe, to the best of our knowledge, no data are available for 2025. In the last complete report in this regard, which dates back to 2019, “the proportion of neuroinvasive infections among infections with known clinical manifestation (67%) was slightly higher than in 2018 (64%). The case fatality in 2019 was also slightly higher than in 2018 (12% compared to 11%)” [81]. Again, since it is well known that neuroinvasive forms are a small proportion of the total cases, these data provide indirect evidence of a very relevant underreporting of WNV cases in Europe. The lack of registration of most asymptomatic/paucisymptomatic cases may hinder the understanding of the disease progression and the individuation of genetic and/or environmental factors putatively predisposing to or protecting against severe forms of the disease. Furthermore, this may hamper timely activation of clinical trials, constrain patient recruitment during short and seasonal transmission windows, and reduce incentives for sustained investment in antiviral and vaccine development. As a result, gaps in surveillance contribute not only to delayed public health responses but also to the persistent lag in translating preclinical antiviral candidates into clinically actionable therapies.

### 2.4. Future Projections and Public Health Implications

The increasing WNV incidence along with progressive population aging creates a major emerging public health challenge. Climate projection models estimate a potential five-fold increase in WNV outbreak risk across Europe by 2040–2060 compared with that in 2000–2020. As previously mentioned, climatic factors play a critical mechanistic role in shaping WNV transmission dynamics. Increases in summer temperatures significantly shorten the extrinsic incubation period in Culex pipiens, thereby accelerating viral amplification, while drought and rainfall anomalies exert nonlinear effects on vector abundance by altering larval habitats and forcing avian hosts to aggregate in limited wetlands. Extreme climatic events and milder winters enhance overwintering survival of diapausing females, prolonging the transmission season [83,84,85,86,87,88]. These eco-climatic processes, combined with anthropogenic land-use changes and reduced avian biodiversity, create increasingly favorable conditions for sustained WNV circulation across Europe, and deserve greater attention. Indeed, current WNVI case counts almost certainly represent only the visible portion of a much larger reservoir of infection. Given the favorable climatic conditions for WNV transmission in Europe, the number of human cases and outbreaks is expected to rise. Recent projection models estimate a potential five-fold increase in WNV outbreak risk by 2040–2060, depending on geographic area and climate scenario, compared with that in 2000–2020 [89]. The proportion of Europeans at risk may rise from 15% to 23–30%, corresponding to 161–244 million people, with Western Europe predicted to experience the largest increase in outbreak probability [90,91].

At the same time, the number of Europeans aged 65 and older is expected to rise to 30% by 2050, with the largest growth happening in the oldest age groups (≥80 years), who are at the greatest risk of serious outcomes. This demographic–epidemiological convergence indicates that even steady age-specific attack rates will result in significantly elevated absolute numbers of older adults suffering life-threatening neuroinvasive diseases. Geographic diversity in viral transmission and population aging exacerbates these issues. Southern and Eastern European areas that are currently seeing the most WNV spread are also seeing their populations age quickly. On the other hand, Western European countries that are currently seeing less WNV spread but have populations that are aging quickly may see the biggest relative increases in geriatric cases as the virus spreads westward [85,92]. Urban–rural differences in both healthcare access and exposure risk create additional complexity, as older residents of rural areas may face higher mosquito exposure but have limited access to specialized neurological care [15].

The epidemiological scenario is additionally complicated by the frequent under-recognition of WNV disease in older individuals. The EFSA December 2025 report explicitly acknowledges that “owing to delays in diagnosis and reporting, as well as the fact that most of the WNV infections are asymptomatic or subclinical, the case numbers provided in this report likely underestimate the true number of cases” [77]. However, several additional factors may contribute to WNV under-recognition in geriatric patients. First, despite asymptomatic infections representing the vast majority of WNVIs, seroprevalence studies in older individuals are limited. Second, WNVI diagnosis in older individuals may be missed because of atypical presentations, delayed healthcare seeking, and impaired antibody production. Third, cognitive impairment and communication difficulties may delay recognition of early neurological symptoms. Fourth, older individuals in nursing homes or assisted living facilities may face barriers to timely medical evaluation during outbreak periods. Finally, the seasonal surveillance predominantly captures severe cases requiring medical attention, missing milder infections that may nonetheless cause significant morbidity in older frail individuals [77]. These epidemiological realities underscore the urgent need for age-specific pharmaceutical interventions. Such interventions must account for age-related pharmacokinetic and pharmacodynamic changes, altered immune responses in older individuals, and the high prevalence of comorbidities and polypharmacy in this population. The concentration of 70% of 2025’s European WNV burden in Italy alone, predominantly affecting older residents of specific high-transmission regions, makes it an ideal setting for targeted trials of age-appropriate pharmaceutical interventions.

## 3. Therapeutic Approaches to WNVI in General and Geriatric Populations

### 3.1. Standard Clinical Management

As of now, there is no specific antiviral medicine or human vaccination for WNVI, and supportive care is still the major way to treat acute WNND. Standard care includes intravenous fluids for hydration, respiratory support (including mechanical ventilation when needed), sedatives for agitation or discomfort, analgesics for pain control, anticonvulsants for seizure management, and corticosteroids in selected severe cases to reduce cerebral oedema (Table 1) [93].

The management of WNND in older patients presents unique challenges that require careful consideration of age-related physiological changes. Age-related decline in cardiac and renal function may increase the risk of fluid overload and electrolyte disorders, especially when fluid management is not adequately performed. The need for and duration of mechanical ventilation for respiratory support should consider the higher risk of ventilator-associated pneumonia in this population; anticonvulsants, sedatives and analgesics must be carefully titrated to avoid oversedation, delirium, and respiratory depression. The high prevalence of polypharmacy in older populations necessitates thorough medication reconciliation to identify and manage potential drug–drug interactions that could exacerbate neurological symptoms or interfere with supportive care [13,93].

The efficacy and safety profiles of corticosteroid agents in the treatment of neuroinvasive forms of WNV encephalitis remain uncertain and subject to ongoing debate, as current evidence is limited to some case reports. One of them described a significant clinical improvement in a 61-year-old man with WNV encephalitis after a five-day course of high-dose corticosteroids [95]. Another 71-year-old man with WNND experienced rapid neurological recovery after a receiving high-dose corticosteroid therapy [94]. Although intriguing, these findings remain anecdotal and insufficient to recommend routine use of corticosteroids. Corticosteroids are used to decrease inflammatory cerebral edema and modulate excessive immune responses; however, their benefits must be weighed against potential risks, especially in older individuals, characterized by increased risk of immunosuppression and susceptibility to infections, impaired wound healing, higher risk of hyperglycemia, gastrointestinal bleeding, psychosis, and osteoporotic fractures [96]. Due to the lack of research trials assessing the efficacy and safety of corticosteroids in older WNVI cases, the choice to administer these drugs to older patients should be individualized.

### 3.2. Experimental Antiviral Candidates

While there is currently no licensed antiviral treatment for WNV, various drugs have shown the ability to limit viral replication in experimental settings (preclinical evidence), while only a limited number have progressed to human evaluation (early clinical evidence), awaiting confirmation through dedicated randomized clinical trials (Table 2). The development of effective antivirals for WNND encounters significant challenges, such as the necessity for blood–brain barrier penetration [97], the attainment of therapeutic concentrations in infected neural tissue [98], and the establishment of safety profiles suitable for administration to older patients with multiple comorbidities [13].

#### 3.2.1. Small Molecule Inhibitors with In Vitro Activity

Several small molecule inhibitors, including cilnidipine, mycophenolate mofetil, nitazoxanide, and teriflunomide, have demonstrated inhibitory activity against WNV replication in Vero cells and SH-SY5Y neuroblastoma cells [99]. Tang et al. identified these clinical candidates through systematic activity screening in vitro followed by effect evaluation in vivo, establishing a foundation for potential therapeutic repurposing [99].

Cilnidipine, a calcium channel blocker used for hypertension, may suppress viral entry by inhibiting membrane fusion and modulating host signaling pathways [99]. For older patients, cilnidipine may offer theoretical advantages based on its use in the treatment of hypertension, though antiviral efficacy remains preclinical [110]; furthermore, a previous study on the effect of the drug against influenza A virus has shown that the dose required for antiviral activity may differ substantially from standard antihypertensive dosing [111]. This may imply the need for careful evaluation of hemodynamic effects in older patients with reduced cardiovascular reserve in view of using this drug in WNV disease.

Mycophenolate mofetil is widely used as an immunosuppressant in organ transplantation; it inhibits inosine monophosphate dehydrogenase (IMDPH), thereby blocking guanosine synthesis required for nucleic acid production in both immune cells and viruses [99]. Similarly, its active metabolite, mycophenolic acid, has been shown to suppress WNV replication in Vero cells [100], but its immunosuppressive effects raise concerns for clinical applicability, particularly in older individuals, as they may facilitate viral dissemination and neuroinvasion.

Nitazoxanide is an antiparasitic medication known also for its broad-spectrum antiviral properties; it activates innate antiviral responses by stimulating the production of type I interferon (IFN-α/β) by fibroblasts [99]. This medication does not have immunosuppressive properties and may give benefits to older patients with weakened immunity, although no human data in WNV are available.

Teriflunomide, which is licensed to treat multiple sclerosis, stops dihydro-orotate de-hydrogenase, which lowers the production of pyrimidines and blocks activated lymphocytes from growing without directly killing cells [99,112]. This drug has immunomodulatory properties, but its relevance to WNV remains preclinical; it is metabolized by the liver and can cause elevation in transaminases, so caution is needed in patients with decreased hepatic reserve.

#### 3.2.2. Nucleoside Analogues

Among the most important nucleoside analogues tested for WNVI, ribavirin is characterized by broad-spectrum antiviral activity and was proposed for WNV treatment based on its in vitro capacity to inhibit viral replication. Ribavirin suppresses WNV through two mechanisms, lethal mutagenesis and inosine 5′-monophosphate dehydrogenase (IMDPH) inhibition, thus decreasing guanine nucleotide biosynthesis. However, during a large human WNV outbreak occurring in Israel in 2000, ribavirin use was associated with increased mortality [102]; the Infectious Diseases Society of America subsequently recommended against ribavirin use for human WNVI based on this clinical evidence [101].

Remdesivir, a monophosphate prodrug of a modified adenosine analogue initially designed for Ebola virus and subsequently approved as the first antiviral treatment for Coronavirus disease 2019 (COVID-19), displays broad activity against multiple RNA viruses and efficiently inhibits flavivirid polymerases [103,104,105,106,107]. Notably, during a recent WNV outbreak in the Campania region, Italy, four patients treated off-label with remdesivir showed clinical improvement and reduced length of hospital stay, with deaths limited to two individuals with concomitant bacterial infections, suggesting that remdesivir might be a potential candidate for therapeutic repurposing in WNV encephalitis, but further studies will be necessary to confirm these preliminary observations [113]. This drug has been shown to be effective and safe in older adults with COVID-19, though monitoring for hepatic and renal adverse effects remains essential, and efficacy in WNVI must be confirmed in randomized controlled trials. Remdesivir’s intravenous formulation ensures reliable bioavailability regardless of age-related changes in gastrointestinal absorption. The drug’s mechanism, based on the direct inhibition of viral polymerase without primary immunosuppressive effects, avoids immunological concerns that limit ribavirin and mycophenolic acid. However, reduced renal function necessitates careful assessment of creatinine clearance and potential dose adjustment to prevent accumulation of the cyclodextrin excipient associated with renal toxicity.

Sofosbuvir, a nucleotide analogue approved for hepatitis C virus (HCV) infection, has also been identified as a potential inhibitor of WNV NS5, consistent with the high degree of structural homology among flaviviral polymerases [114,115]. In vitro and preclinical studies have demonstrated broad anti-flaviviral and anti-alphaviral activity, including inhibition of Zika, chikungunya, and more recently WNV replication [116,117,118,119]. However, so far there is only a single study assessing the in vitro efficacy of sofosbuvir against WNV; this investigation reported on one hand effective anti-WNV replication activity [116], but on the other hand detected the S604T mutation able to confer antiviral resistance. Taken together, these preliminary in vitro findings suggest that sofosbuvir may be a potential repurposing candidate within combination regimens designed to limit resistance emergence [116].

#### 3.2.3. Interferon-Based Therapies

IFN-I constitutes a central component of the innate immune response against WNV and limits both viral replication and neuroinvasion in experimental models [120]. In vitro, IFN-α, IFN-β, and IFN-ω all limit WNVI, with IFN-β displaying the most potent antiviral activity, particularly in retinal and epithelial cell lines [121]. Clinically, IFN-α has been used in isolated cases of WN encephalitis with anectodical neurological improvement reported in isolated case reports [108], but robust evidence is lacking. Recent immunological studies have shown that approximately 40% of patients with WNV encephalitis harbor neutralizing autoantibodies against IFN-α and IFN-ω, whereas IFN-β is rarely targeted [109,122,123]. The presence of IFN-I autoantibodies correlates with more severe disease, suggesting that early therapeutic administration of IFN-β might bypass this neutralization. With this background, a randomized, double-blind, placebo-controlled trial is currently evaluating IFN-β1a to prevent progression to WNND and improve outcomes in patients with acute WNVI (NCT06510426) [85].

#### 3.2.4. Peptide-Based Inhibitors and Monoclonal Antibodies (mAb) Against Envelope (Ep) Proteins

Intensive research has focused on the individuation of viral epitopes representing a valid putative target for drug development. In this framework, several antiviral candidates targeting the WNV envelope protein (Ep) or NS protein [124] have been proposed. The most significant compounds targeting structural and NS proteins are reported in Table 3.

In this regard, it is important to keep in mind that the Ep, located on the virion surface, is essential for viral attachment and entry and is a major target of neutralizing antibodies. However, the Ep contains highly conserved epitopes, particularly the fusion loop (FL), shared across multiple flaviviruses. These epitopes may elicit cross-reactive antibodies that may enhance infection and disease severity by heterologous flaviviruses by means of the antibody-dependent enhancement of infection (ADE) phenomenon, rendering FL-based vaccine approaches unsuitable [136]. For older European populations, ADE risk requires consideration given the widespread prior exposure to related flaviviruses. Many older adults in endemic regions may harbor antibodies from previous dengue virus exposures during travel, yellow fever vaccination, tick-borne encephalitis, or even prior asymptomatic WNVI. Cross-reactive antibodies from these exposures could theoretically enhance subsequent WNVI if therapeutic antibodies administered to the patient bind the same cross-reactive epitopes without providing robust neutralization. This concern mandates that any E-protein-targeted therapeutics for older patients must be evaluated for potential ADE effects in preclinical models using sera from individuals with diverse flavivirus exposure histories [136]. Conversely, humanized mAbs targeting the Ep have demonstrated some efficacy in animal models only, despite CNS delivery remaining challenging. For instance, the Ep16 mAb inhibited WNVI by 50% in hamsters, achieving survival rates of 80% after intraperitoneal administration and 88% when administered intracerebrally [125]. These findings suggest that mAbs may neutralize WNV even within the CNS, likely aided by BBB disruption during neuroinflammation [125]. Limitations of mAbs challenges in achieving therapeutic CNS concentrations are associated with an age-dependent increase in BBB permeability, with subsequent unpredictable intracerebral pharmacokinetics. Emerging experimental approaches to overcome this limitation include Fc-engineering to enhance FcRn-mediated transcytosis across the BBB [137], the use of nanocarriers or extracellular vesicles as delivery systems [138,139], and intranasal administration to exploit nose-to-brain transport pathways [140]. Despite these innovative approaches, a fundamental limitation of mAb therapeutics for WNV is its high production costs; mAb manufacturing requires sophisticated bioreactor facilities, extensive quality control, and cold-chain distribution, which may limit availability during outbreaks in resource-limited regions. The cost per treatment course typically ranges from thousands to tens of thousands of dollars, potentially unaffordable for affected patients in many European countries even with insurance coverage. Furthermore, the requirement for IV administration (unless intranasal delivery proves viable) necessitates hospitalization or infusion center visits, creating access barriers for older frail patients.

Also non-conserved Ep regions exhibit therapeutic potential [141]. For example, four cyclic peptides (CTKTDVHFC, CIHSSTRAC, CTYENHRTC, CLAQSHPLC) targeting the DIII receptor-binding domain have demonstrated in silico affinity and in vitro neutralizing activity without triggering ADE. Peptide P1 (DTRACDVIALLCHLNT) and its derivative P9 (CDVIALLACHLNT) inhibited WNVI in vitro; moreover, P9 reduced viremia and brain viral load, crossed the BBB, and improved survival in murine models [126]. Administration of anti-Ep peptides to older patients requires weighing advantages and risks; the advantages include their small size which facilitates them to easily cross the blood–brain barrier; additionally, their target-specific action prevents off-target effects and drug–drug interactions; finally, their administration is not associated with risk of ADE. On the other hand, potential limits of these molecules include their short half-life, which requires frequent IV administration, and their high cost, which makes the treatment unaffordable for patients with limited vascular access or economic constraints.

#### 3.2.5. Peptide-Based Inhibitors Against NS Proteins

Medications targeting NS protein 1 (NS1), which is involved in replication, immune evasion, and tissue damage, might produce antiviral actions. Indeed, experimental evidence has shown that anti-NS1 antibodies are protective in mice [142]. Although NS1 is absent in virions, the protection is induced through multiple mechanisms: the blockage of NS1-mediated immune evasion, prevention of NS1-induced endothelial damage and vascular leak, decrease in NS1-mediated complement activation, and potentially facilitation of antibody-dependent cellular cytotoxicity (ADCC) against infected cells expressing NS1 on their surface [142]. As such, administration of anti-NS1 peptides to older patients may be useful because of the strain specificity and low ADE risk; however, their ability to cross the blood–brain barrier is uncertain.

Other NS proteins, particularly NS3 and NS5, represent attractive drug targets (Table 3). NS3 protease cleaves the viral polyprotein into functional units and is essential for viral replication. Multiple experimental approaches have explored NS3 inhibition [143,144,145]. Zafirlukast and its derivatives bind an NS3 allosteric site and inhibit protease activity via in vitro and in silico analyses [127]. Among NS3 inhibitors, Cbz-Lys-Arg-(4-GuPhe)P(OPh)_2_ is especially potent [128]. Furthermore, xanthine-core compounds have emerged as a promising class of NS2B-NS3 protease inhibitors with demonstrated antiviral activity in mouse and human microsomes, though limited by cytotoxicity, low solubility, and modest metabolic stability [129]. Other compounds identified through high-throughput screening, including tolcapone, tannic acid, and a catechol-based “compound C”, competitively bind the NS3 active site [130]. Nevertheless, tolcapone, despite its inhibitory properties, is contraindicated in liver disease due to known hepatotoxicity. Additional NS3 inhibitors include eugenol derivatives [146] and various peptide-β-lactams [131]. Metallopeptides such as amino-terminal copper- and nickel-binding peptides inhibit NS3 by oxidizing catalytic and substrate-binding residues, thereby attenuating protease activity [132].

NS5, another crucial viral enzyme involved in WNV RNA synthesis and methylation, has also been extensively explored as an antiviral target [144,145,147,148,149,150,151,152,153,154]. Cyclophilins are host proteins implicated in numerous cellular processes and appear to be required for WNV replication, although mechanistic details remain incomplete and in vivo data are lacking [133,134]. Small molecules such as tyrphostin AG538 (I-OMe-AG538) and suramin hexasodium can inhibit NS3–NS5 interactions critical for replication, although suramin has not received FDA approval because of adverse effects [135]. Additional inhibitors, including C-9 (ZINC1333392), C-24 (ZINC56798609), and C-30 (ZINC19598270), disrupt NS3–NS5 interactions in a concentration-dependent manner both in vitro and in mouse models, further supporting protein–protein interaction targeting as a promising antiviral strategy [155].

The diversity of antiviral approaches targeting WNV replication highlights the complexity of the viral life cycle and the multiple opportunities for pharmacological intervention. Targeting host factors involved in viral replication and innate antiviral pathways may yield broad-spectrum agents, an advantageous property for future antiviral development. Although many individual inhibitor candidates demonstrate efficacy in vitro, most lack in vivo validation, and many of them have side effects that would make them unsuitable for older patients (Table 3). Combination therapies targeting multiple viral proteins, such as NS3 and NS5, may help prevent escape mutations and enhance antiviral potency. Future research should explore such combinations to advance toward effective therapeutic options for WNV.

## 4. Passive and Active Immunization Strategies Against WNV

### 4.1. Passive Immunization Strategies

Passive immunization has been attempted in a few acute WNVI cases. Although donor antisera can neutralize WNV, this approach carries substantial limitations, including the risk of transmitting blood-borne pathogens, variability in antiserum quality, high cost, and potential hypersensitivity reactions [156]. WNV-specific IVIG (WNIG), produced from donors in endemic areas with high anti-WNV titers, has demonstrated approximately ten-fold higher neutralizing potency than standard IVIG in vitro and superior efficacy in murine models, including 100% survival in dexamethasone-immunosuppressed mice when administered shortly after infection [157]. These data suggest that WNIG could represent an experimental antibody-based approach pending human clinical trials.

### 4.2. Vaccine Development

Although this review focuses primarily on therapeutic interventions for acute disease, the long-term preventive solution to WNV burden in geriatric European populations would rely on successful vaccine development. No WNV vaccine candidate has progressed beyond phase I or II clinical trials, and existing formulations often require multiple doses and boosters [158]. Several vaccine platforms, including inactivated, recombinant subunit, DNA, and viral-vectored candidates, have demonstrated immunogenicity and protective efficacy in preclinical models [158]. Veterinary vaccines for horses are widely used across Europe and North America, demonstrating the feasibility of WNV immunization [159]. The success of equine vaccines, which have dramatically reduced WNV disease in horses, proves that protective immunity against WNV can be safely induced through vaccination [159]. However, translating this success to human vaccines faces substantial challenges, particularly for older populations: age-related immune senescence compromises vaccine responses, with older individuals typically generating lower antibody titers and shorter-lived immune memory compared to younger adults as observed for influenza vaccination [160]. Studies across multiple vaccine platforms (e.g., influenza, hepatitis B, and SARS-CoV-2) show that standard two-dose regimens often fail to achieve durable protection in older adults. Consequently, older populations may require additional doses or periodic boosters to maintain adequate immunity [161,162,163]. For the European older population specifically, a successful WNV vaccine would provide transformative public health benefit. Given the 47% increase in cases above the 10-year average observed in 2025, and climate projections suggesting five-fold increases in outbreak risk by 2060, the window for preventing a looming epidemic in aging European populations is rapidly closing. Accelerated vaccine development specifically targeting older individuals, the population bearing 70–90% of disease burden, should be recognized as an urgent European public health priority requiring coordinated multinational investment and streamlined regulatory pathways.

## 5. Clinical and Translational Challenges Limiting Effective Antiviral Strategies for WNV in Older Adults

Despite substantial progress in delineating viral biology, immunopathogenesis, and genomic diversification, at present, supportive care remains the only clinically actionable treatment for WNND, while all antiviral and immunological strategies remain preclinical or early clinical. Many compounds, including repurposed drugs, have indeed showed potential therapeutic effects against WNV in preclinical investigations, including both in vitro and in animal models studies, but until now, no drug or vaccine has demonstrated a favorable efficacy/safety profile in large randomized clinical trials. There are many factors that may contribute to delayed translation of preclinical drugs into clinically actionable options, especially in older adults, representing the population at highest risk for WNND, long-term disability, and mortality (Table 4).

First, it is not easy to find effective drugs able to cross the BBB in vivo at a sufficient degree, thus blocking the virus-associated neurotoxic effects; on the other hand, the BBB impairment which is typically observed during WNV encephalitis, often exacerbated by age-related endothelial dysfunction and neuroinflammation, may render the cerebral pharmacokinetics of drugs able to cross the BBB unpredictable, thereby complicating dose optimization and safety assessment [164].

Immunological strategies face additional challenges. Passive immunization and monoclonal antibodies against WNV may potentially induce the ADE phenomenon, as cross-reactive but non-neutralizing antibodies may exacerbate disease severity, thus substantially increasing the mortality of possible subsequent flavivirus infections [172]. This risk is especially concerning in older adults living in endemic regions, who may share pre-existing antibodies from prior asymptomatic WNVI, Zika virus infection, tick-borne encephalitis, dengue exposure, or yellow fever vaccination [173,174,175].

Third, some of the repurposed drugs against WNV have immunosuppressant or immunomodulatory effects: this logically implies that these drugs on one hand may inhibit WVN replication and spreading, but on the other hand, they may impair the host immune system; therefore, the net clinical benefit of these drugs is not easy to forecast, especially in older people whose immune system is naturally impaired by immunosenescence, characterized by decreased interferon responses, T-cell function, and dysregulated cytokine production [165].

Fourth, age-related pharmacokinetic and pharmacodynamic changes represent a significant and often overlooked barrier to translation. Decreased renal clearance and hepatic metabolism, as well as changes in body composition, increased blood–brain barrier permeability, and modifications in serum protein binding, may significantly affect drug exposure and toxicity in older patients [166]. These factors may differentially affect major antiviral classes: nucleoside analogues may accumulate in patients with kidney function impairment, monoclonal antibodies may display prolonged half-lives with unpredictable tissue distribution, and interferons may provoke exacerbated systemic and neuropsychiatric adverse effects. The high prevalence of multimorbidity, polypharmacy, and frailty in older adults further increases the risk of clinically significant drug–drug interactions and adverse events, limiting tolerability and feasibility of many candidate therapies in real-word settings [167,168].

Finally, immunosenescence also constrains preventive strategies, as older individuals frequently mount weaker, delayed, and less durable immune responses to vaccination. This phenomenon has been consistently observed across multiple vaccine platforms, including influenza, hepatitis B, and SARS-CoV-2 vaccines [169], and potentially contributes to the limited success of WNV vaccine candidates in this population [176,177]. Collectively, these biological, pharmacological, and structural barriers, combined with the episodic nature of WNV outbreaks, delayed diagnosis, and the absence of coordinated, age-inclusive clinical trial infrastructure, have repeatedly prevented the successful translation of preclinical antiviral efficacy into clinically effective therapies for WNV. Addressing these challenges will require age-specific drug development strategies, incorporation of geriatric pharmacology into early-phase studies, and clinical trial designs that adequately represent the older populations most affected by WNV disease.

## 6. A Potential Europe-Wide Model for Clinical Trial Readiness

The Pharmaceutical Strategy for Europe, adopted on 25 November 2020, seeks to establish a regulatory framework that effectively integrates pharmaceutical innovation with public health infrastructure. Ensuring timely access to safe, effective, innovative, and affordable medicines represents a foundational component of the European health system. To fully exploit the transformative potential of digital technologies, secure and efficient access to high-quality health data is essential. In this context, epidemiological metadata collected by ECDC, including monthly updates on human and animal WNVI cases with known places of infection, provides a crucial foundation for coordinated response strategies.

Despite these advances, no European Union-wide model for clinical trial readiness specific to WNVI has yet been established. Developing such a model is essential to accelerate therapeutic development and ensure rapid deployment of clinical studies during seasonal outbreaks.

We propose a two-pronged strategy to enhance Europe’s preparedness to face future WNV epidemics:

- Systematic screening of existing antiviral and immunomodulatory agents with established safety profiles to identify those with WNV inhibitory activity, leveraging in vitro platforms and advanced in silico technologies.
- Creation of a European clinical trial network capable of rapidly initiating adaptive therapeutic studies during seasonal outbreaks, ensuring timely evaluation of promising antiviral candidates.

A comprehensive EU model for WNV infections should integrate multiple categories of data:

- Environmental variables, which influence avian reservoir dynamics and mosquito population behavior, particularly that of *Culex pipiens*, as well as seasonal variations across European regions.
- Biotic and ecological factors affecting vector distribution, abundance, and WNV transmission dynamics.
- Genomic data, including WNV sequencing across Europe, to clarify transmission patterns, support outbreak investigation, and reconstruct the spatiotemporal spread of circulating strains. In this regard, it is important to underline that genomic analysis has significantly expanded understanding of WNV epidemiology within Europe, and a global Nextstrain dataset of WNV genomes is publicly available [90].
- Human and avian immunologic response data, which reflect levels of vector exposure and local transmission intensity, supporting more targeted and effective surveillance strategies.
- Pharmaceutical development datasets, such as those provided by GlobalData [178], which summarize mechanisms of action for antiviral candidates, products in clinical development, agents undergoing regulatory review, post-authorization data, and updates on key industry stakeholders.

In addition to routine pharmacovigilance, post-authorization platforms are needed to monitor the safety and real-world effectiveness of novel WNV therapeutic strategies. In this context, different European infrastructures, especially those involved in pharmacovigilance, should allow continuous data integration and rapid detection of safety signals as new agents are deployed. By improving the connection between pre-clinical pharmaceutical research and existing public health surveillance systems, the European Union may shorten the translational gap from drug discovery to clinical application, thus reinforcing outbreak response capacity and enhancing preparedness for future WNV epidemics. This integrated, data-driven model represents a critical step toward a coordinated, resilient European strategy for managing emerging vector-borne threats.

To illustrate how the proposed trial-readiness framework could function in practice, a hypothetical seasonal outbreak scenario can be considered. During the early summer period, routine integrated surveillance identifies a sustained increase in WNV-positive mosquito pools and avian mortality in a defined geographical area, followed by the first laboratory-confirmed human neuroinvasive cases reported through national surveillance systems. Once predefined epidemiological and laboratory thresholds are met, an alert is automatically communicated to a pre-established European trial coordination network.

At this stage, a pre-approved, adaptive platform trial protocol—previously reviewed by ethics committees and regulatory authorities—is activated. Participating clinical sites in affected regions are rapidly notified, and patient screening begins immediately upon hospital admission for suspected WNND. Eligible patients are enrolled using harmonized case definitions and electronic case report forms, allowing near-real-time data capture.

Ensuring that older adults are actively included in WNV clinical trials requires trial designs that accommodate, rather than exclude, age-related functional, cognitive, and logistical challenges. Given that WNND predominantly affects older individuals, eligibility criteria should prioritize clinical relevance over artificial restrictions based on comorbidity, polypharmacy, or functional impairment. Hospital-based enrollment at the point of acute care allows inclusion of patients with limited mobility or acute neurological symptoms, while simplified screening procedures facilitate timely recruitment during outbreaks. To address cognitive impairment or acute delirium, adaptive consent models, such as deferred consent, proxy consent, or re-consent once capacity is regained, can be implemented in accordance with national regulations and ethical guidance. Follow-up procedures may be decentralized and flexible, incorporating home visits, telemedicine, or structured telephone assessments to minimize travel burden and loss to follow-up. Importantly, outcome measures should reflect geriatric priorities, including functional status, quality of life, and recovery trajectories, rather than relying solely on short-term virological endpoints. Together, these strategies shift the focus from passive inclusion to deliberate trial designs that reflect the clinical realities of older adults, thereby improving both ethical validity and the generalizability of evidence generated during seasonal WNV outbreaks.

The adaptive design enables early interim analyses, permitting ineffective treatment arms to be discontinued and promising candidates to be expanded or newly introduced as the outbreak evolves. If transmission spreads to additional regions, further sites are activated without requiring protocol redesign. Conversely, if case numbers decline at the end of the transmission season, enrollment is paused while maintaining the infrastructure for reactivation in subsequent seasons.

This workflow demonstrates how advance planning, harmonized surveillance triggers, and adaptive trial designs could transform episodic WNV outbreaks into predictable opportunities for efficient clinical research, thereby accelerating the generation of robust evidence and reducing reliance on small case series or compassionate-use data.

## 7. Conclusions

WNV has evolved from a traditionally neglected tropical pathogen into a significant public health threat worldwide, driven by accelerating climatic shifts, ecological perturbations, and expanding vector and reservoir populations. The increasing frequency, geographic range, and severity of outbreaks underscore the widening gap between the epidemiological burden of WNND and the limited availability of effective pharmaceutical strategies in older adults.

Given these constraints, Europe urgently needs a coherent pharmaceutical preparedness plan tailored to WNV. We propose a two-pronged strategy: (i) systematic screening and repurposing of antiviral and immunomodulatory agents with established safety profiles, leveraging advanced computational approaches, high-throughput in vitro platforms, and multi-omics integration; and (ii) the establishment of a scalable, interoperable European clinical trial network capable of rapid activation during seasonal outbreaks. Such a platform, embedded within a strengthened One Health surveillance architecture and linked to genomic, ecological, and immunological datasets, would markedly reduce translational delays, enable adaptive trial designs, and facilitate real-time pharmacovigilance and post-authorization monitoring.

## Figures and Tables

**Table 1:** Clinical utilization of supportive care strategies in older patients with WNVI.

Drug/Care	Mechanism of Action	Evidence	Geriatric Considerations
IV fluids [93]	Hydration and hemodynamic support	Standard of care	Risk of fluid overload in older patients with cardiac/renal dysfunction, careful monitoring is essential.
Mechanical ventilation [93]	Respiratory support	Standard of care	Higher risk of VAP, difficulty weaning; balance need vs. risks.
Anticonvulsants [93]	Seizure control	Standard of care	Dose adjustment for reduced renal clearance; increased CNS sensitivity in older individuals.
Analgesics and/or sedatives [93]	Pain/agitation management	Standard of care	Risk of oversedation, delirium, respiratory depression; careful titration required.
Corticosteroids [93]	Reduce cerebral edema	Case reports [94,95]	Increased infection risk in immunosenescent older patients; metabolic complications (hyperglycemia, etc.); no RCT evidence.

Notes: CNS = central nervous system; IV = intravenous; RCT = randomized clinical trial; VAP = ventilator associated pneumonia.

**Table 2:** Mechanism-based antiviral therapeutic agents for WNVI in older patients.

Drug/Care	Mechanism of Action	Evidence	Potential Implications for Older Adults
**Small molecule antivirals**			
Cilnidipine [99]	Inhibits viral entry	Preclinical	Established cardiovascular safety in hypertensive older individuals; antiviral dose may differ from antihypertensive dose.
Mycophenolate mofetil and mycophenolic acid [99,100]	Inhibit guanosine synthesis	Preclinical	Immunosuppressive effects concerning immunosenescent older individuals; may impair viral clearance.
Nitazoxanide [99]	Enhances IFN response	Preclinical	Immune-stimulating rather than suppressive; may benefit older patients with compromised immunity.
Teriflunomide [99]	Reduces pyrimidine synthesis	Preclinical	Immunomodulatory vs. immunosuppressive; liver enzyme monitoring required.
**Nucleoside analogues**			
Ribavirin [101,102]	Induces lethal mutagenesis	Early negative clinical	Associated with mortality in recent outbreak; risk of hemolytic anemia.
Remdesivir [103,104,105,106,107]	Inhibits RNA polymerase	Limited early clinical data from case series	Extensive COVID-19 safety data in older patients; IV formulation ensures bioavailability; renal monitoring needed.
Sofosbuvir [102]	Inhibits RNA polymerase	Preclinical	Oral bioavailability; extensive HCV safety data in older adults; resistance concerns suggest combination use.
**Interferon-based therapies**			
IFN-α [108,109]	Enhances antiviral response	Limited early clinical data from case reports	Neutralized by autoantibodies in ~40% of patients (higher in older individuals); limited efficacy.
IFN-β1a [85]	Enhances antiviral response	Phase III RCT ongoing (NCT06510426)	Rarely targeted by autoantibodies; may overcome neutralization in older individuals; trial should include age-stratified analysis.

Notes: IFN = interferon; RCT = randomized clinical trial.

**Table 3:** Candidate antiviral therapies for WNVI: clinical applicability and geriatric considerations.

Drug/Care	Mechanism of Action	Evidence	Geriatric Considerations
**Inhibitors of envelope (e) proteins**			
Humanized Ep16 mAb [125]	Viral neutralization	Preclinical	CNS penetration challenges; high cost; may benefit from Fc-engineering or intranasal delivery.
Cyclic peptides (E protein DIII-targeting) [126]	Block receptor binding	Preclinical	Small size may facilitate BBB crossing; no ADE risk with non-conserved epitopes.
P9 peptide [126]	Block viral entry	Preclinical	Demonstrated BBB crossing in mice; short half-life may require frequent dosing.
**NS2B-NS3 protease inhibitors**			
Zafirlukast [127]	Allosteric inhibition	Preclinical	Established asthma safety in older patients; antiviral dose unknown.
Cbz-Lys-Arg-(4-GuPhe)P(OPh)_2_ [128]	Active site inhibition	Preclinical	Most potent NS3 inhibitor identified; no safety data.
Xanthine-core compounds [129]	Protease inhibition	Preclinical	Limited by cytotoxicity, solubility issues; needs optimization.
Tolcapone [130]	Competitive inhibition	Preclinical	Hepatotoxicity risk unacceptable in older patients with hepatic compromise.
Peptide-β-lactams [131]	Dual binding modes	Preclinical	No safety/PK data.
ATCUN metallopeptides [132]	Oxidative inhibition	Preclinical	Novel mechanism development.
**NS5/host-directed agents**			
Cyclophilin inhibitors [133,134]	Block NS5-cyclophilin interaction	Preclinical	Cyclosporine is immunosuppressive/nephrotoxic; unsuitable for older patients; non-immunosuppressive derivatives needed.
Tyrphostin AG538, Suramin [135]	Block NS3-NS5 interaction; disrupt replication complex	Preclinical	Suramin: significant toxicities (coagulopathy, renal) unacceptable in older patients.
C-9, C-24, C-30 (ZINC compounds)	Block NS3-NS5 interaction	Preclinical	Most advanced PPI inhibitors; oral bioavailability potential; needs safety evaluation in aged models.

Notes: CNS = central nervous system; NS = non-structural.

**Table 4:** Age-related biological and clinical factors limiting the effectiveness of antiviral strategies for WNV in older adults.

Domain	Age-Related Modification	Potential Effects on Treatments for WNV
Blood–brain barrier [164]	Age-related dysfunction and neuroinflammation	Limited or unpredictable CNS drug penetration
Immunosenescence [165]	Decreased interferon responses; impaired T- and B-cell function; chronic inflammation	Decreased antiviral efficacy; unpredictable immune modulation
Decreased renal clearance [166]	Declining GFR; occult CKD	Drug accumulation and toxicity
Decreased hepatic metabolism [166]	Decreased hepatic reserve; enzyme inhibition	Hepatotoxicity; altered drug exposure
Polypharmacy [167]	Multiple concomitant medications	Increased drug–drug interactions
Frailty, multimorbidity [167,168]	Reduced physiological reserve	Narrow therapeutic window; poor tolerability
Vaccine hyporesponsiveness [169]	Blunted antibody responses	Reduced preventive efficacy
Trial representation [170,171]	Under-enrolment of older adults	Limited generalizability to older adults

Notes: CKD = chronic kidney disease; CNS = central nervous system; GFR = glomerular filtration rate.

## Data Availability

No new data were created or analyzed in this study. Data sharing is not applicable to this article.

## Abbreviations

The following abbreviations are used in this manuscript:

ADE	Antibody-Dependent Enhancement
CKD	Chronic Kidney Disease
CNS	Central Nervous System
CSF	Cerebrospinal Fluid
Ep	Envelope
GFR	Glomerular Filtration Rate
IFN	Interferon
IV	Intravenous
NS	Non-structural
VAP	Ventilator-Associated Pneumonia
WNND	West Nile Neuroinvasive Disease
WNVI	West Nile Virus Infection
WNV	West Nile Virus
